# Regional Variation in Androgen Receptor Expression and Biomechanical Properties May Contribute to Cryptorchidism Susceptibility in the LE/orl Rat

**DOI:** 10.3389/fendo.2018.00738

**Published:** 2018-12-04

**Authors:** Joshua T. Morgan, Alan K. Robbins, Abigail B. Mateson, Kazuki Sawamoto, Shunji Tomatsu, Dione R. Gray, Jason P. Gleghorn, Julia Spencer Barthold

**Affiliations:** ^1^Department of Bioengineering, University of California, Riverside, Riverside, CA, United States; ^2^Nemours Biomedical Research, Division of Urology, Nemours/Alfred I. duPont Hospital for Children, Wilmington, DE, United States; ^3^Department of Orthopedics, Nemours/Alfred I. duPont Hospital for Children, Wilmington, DE, United States; ^4^Department of Biomedical Engineering, University of Delaware, Newark, DE, United States

**Keywords:** gubernaculum, cryptorchidism, androgens, glycosaminoglycans, hyaluronan, myogenesis, microaspiration, biomechanics

## Abstract

**Background:** The process of testicular descent requires androgen and insulin-like 3, hormones secreted by fetal Leydig cells. Knowledge concerning distinct and common functions of these hormones in regulating development of the fetal gubernaculum remains limited and/or conflicting. The current studies were designed to better define characteristics of androgen receptor (AR) expression, function and regulation, as well as the biomechanical properties of normal and cryptorchid gubernaculum during fetal development.

**Methods:** We studied fetal gubernacula from Long Evans outbred (LE/wt) rats and an inbred (LE/orl) strain with an inherited form of cryptorchidism associated with an AR signaling defect. Gubernacular cells or whole organs obtained from LE/wt and LE/orl fetal gubernacula underwent AR immunostaining and quantitative image analysis. The effects of dihydrotestosterone (DHT) on AR expression, muscle fiber morphology, hyaluronan (HA) levels and glycosaminoglycan (GAG) content were measured in LE/wt gubernacula. Finally, the spatial mechanics of freshly harvested LE/wt and LE/orl fetal gubernacula were compared using micropipette aspiration.

**Results:** AR is expressed in the nucleus of mesenchymal core, tip and cord cells of the embryonic (E) day 17 and 21 fetal gubernaculum, and is enhanced by DHT in primary cultures of gubernacular mesenchymal cells. Enhanced AR expression at the tip was observed in LE/wt but not LE/orl gubernacula. In *in vitro* studies of whole mount fetal gubernaculum, DHT did not alter muscle fiber morphology, HA content or GAG production. Progressive swelling with reduced cellular density of the LE/wt gubernaculum at E19–21 was associated with increased central stiffness in LE/wt but not in LE/orl fetuses.

**Conclusions:** These data confirm nuclear AR expression in gubernacular mesenchyme with distal enhancement at the tip/cord region in LE/wt but not LE/orl rat fetuses. DHT enhanced cellular AR expression but had no major effects on muscle morphology or matrix composition in the rat fetal gubernaculum *in vitro*. Regional increased stiffness and decreased cell density between E19 and E21 were observed in LE/wt but not LE/orl fetal gubernacula. Developmental differences in cell-specific AR expression in LE/orl fetal gubernacula may contribute to the dysmorphism and aberrant function that underlies cryptorchidism susceptibility in this strain.

## Introduction

Cryptorchidism or undescended testis, is the most common reproductive congenital anomaly (1, 2). Testicular descent normally occurs prior to birth in humans and requires developmental programming of the fetal gubernaculum, an organ comprising a central core of mesenchyme infiltrated and surrounded by cremaster muscle. Studies in rodents indicate that development and function of the fetal gubernaculum are regulated by the Leydig cell hormones insulin-like 3 (INSL3) and androgens via their receptors, relaxin/insulin-like family peptide receptor 2 (RXFP2) and the androgen receptor (AR).

Despite a wide range of studies in mammalian models, not all aspects of the hormonal control of testicular descent are fully understood. A prevailing view, first proposed by Hutson over 30 years ago (3), defines descent as a process that occurs in two separate stages, Phase 1/transabdominal, and Phase 2/transinguinal descent, each regulated by distinct hormones. Transgenic studies confirm that cryptorchidism is present in mice with conditional ablation of either *Insl3, Rxfp2* or *Ar* (the GU-ARKO mouse) in the gubernaculum (4, 5). However, the effects of knockdown or deletion of *Insl3* or *Rxfp2* are more profound than those of *Ar* deletion on gubernacular swelling and testicular descent. With partial knockdown of *Rxfp2*, the murine fetal gubernaculum is disorganized, with aberrant muscle cells in the mesenchymal core, while *Rxfp2* or *Insl3* deletion results in an atrophic and disorganized fetal gubernaculum (4, 6–9). In contrast, cryptorchidism in the GU-ARKO mouse is associated with more subtle gubernacular defects (5) that include abnormal cremaster muscle development, which was also reported in the *Tfm* mouse, a strain with an *Ar-*inactivating mutation (10).

The role of androgens in gubernacular development and the target(s) of androgen action in testicular descent remain controversial. Hutson et al. reported absence of AR expression within the fetal gubernaculum, and hypothesized that prenatal androgens act indirectly via masculinization of the genitofemoral nerve (GFN) (11). Most recently they have proposed that androgens exert indirect effects on tissues extrinsic to the gubernaculum, including stimulation of AR-expressing cells in the inguinoscrotal fat pad and repression of the mammary branch of the GFN (12, 13). In contrast, other investigators have observed AR expression in fetal rodent gubernacular mesenchyme (14–16).

The functional role(s) of AR in testicular descent, in addition to cellular proliferation (17, 18), have been hypothesized to include myogenesis and regulation of extracellular matrix (ECM) composition, although there is little direct evidence in support (4, 5). ECM production is likely important in determining the size and biomechanical properties of the gubernaculum. Additionally, there is supportive evidence of a role for the neuropeptide calcitonin gene-related peptide (CGRP) release by gubernacular sensory nerves, providing non-hormonal stimulation of cellular proliferation and motility in the gubernaculum (11). Studies of rat gubernacula exposed to antiandrogens and of *Tfm* mouse gubernacula suggest a role for neuro-hormonal interaction between androgens and CGRP in the process of testicular descent (19, 20).

To more clearly define the role of AR in gubernacular development, we studied the effects of dihydrotestosterone (DHT) stimulation on AR expression, muscle fiber morphology, and ECM composition, and we compared the pattern of AR expression and the biomechanical properties of the gubernaculum in outbred and LE/orl fetal rats with evidence for impaired AR signaling (21, 22). Prior work has shown the inbred LE/orl strain is susceptible to cryptorchidism, with only 45% of LE/orl fetuses exhibiting bilateral descent (23). Our data suggest that nuclear AR is expressed in a subset of gubernacular mesenchymal cells, particularly within the tip/cord region, and that androgen regulates expression but not muscle size or ECM production in the fetal rat gubernaculum. Although our data suggest that ECM composition is not altered in the LE/orl fetal gubernaculum, we found regional differences in AR expression, cellular density and organ stiffness that are consistent with previous evidence of dysmorphology and may contribute to gubernacular malfunction in this strain.

## Materials and Methods

### Animals and Tissue Samples

We obtained fetal gubernacula from timed-pregnant Long-Evans wild-type (LE/wt) and LE/orl (an LE substrain with inherited cryptorchidism) males by microdissection during Phase 1 (E17 and E19) and Phase 2 (E21) of testicular descent, at the onset of gubernacular inversion. Samples obtained from ≥3 fetuses and ≥2 separate litters were immediately processed for each experiment. All work was conducted under a protocol approved by the Nemours Institutional Animal Care and Use Committee (#NBR-2016-002).

### AR Expression in Fetal Gubernaculum

For tissue clearing we used the benzyl alcohol:benzyl benzoate (BABB) protocol (24) prior to confocal imaging, as described previously (21). Immediately after harvesting, fetal gubernacula were fixed in freshly prepared 4% paraformaldehyde/phosphate buffered saline (PBS) and incubated overnight at 4°C. The gubernacula where then placed in 100% methanol for 1 h on ice followed by storage at −20°C. Samples were then treated with Dents bleach, blocked and incubated overnight with a 1:10 dilution of A4.1025, a myosin-specific mouse monoclonal antibody (Developmental Studies Hybridoma Bank) and 1:200 dilution of a rabbit anti-AR antibody (Abcam cat# ab133273) which we have validated by Western blot to recognize the full length AR protein (data not shown). Gubernacula were then incubated overnight at 4°C, washed 4X with PBS Tween (PBST) and then incubated overnight with donkey anti-rabbit Alexaflour 555 (ThermoFisher Cat#A31572) and DRAQ5 (Themofisher Cat#62254) as a counterstain for nuclei. Gubernacula were then washed 3X for 20 min in PBST and then exposed to 100% methanol on ice and stored at 4°C prior to imaging. Just before imaging, gubernacula were cleared by incubation in BABB (1:2). The LSM880 confocal microscope with 25X oil objective was used to create an image z-stack, with images viewed sequentially to visualize 3-dimensional AR expression within the gubernaculum. Representative single images were in the central sagittal plane were qualitatively reviewed to define developmental AR distribution.

### Quantitative Image Analysis

**Figure 1:**
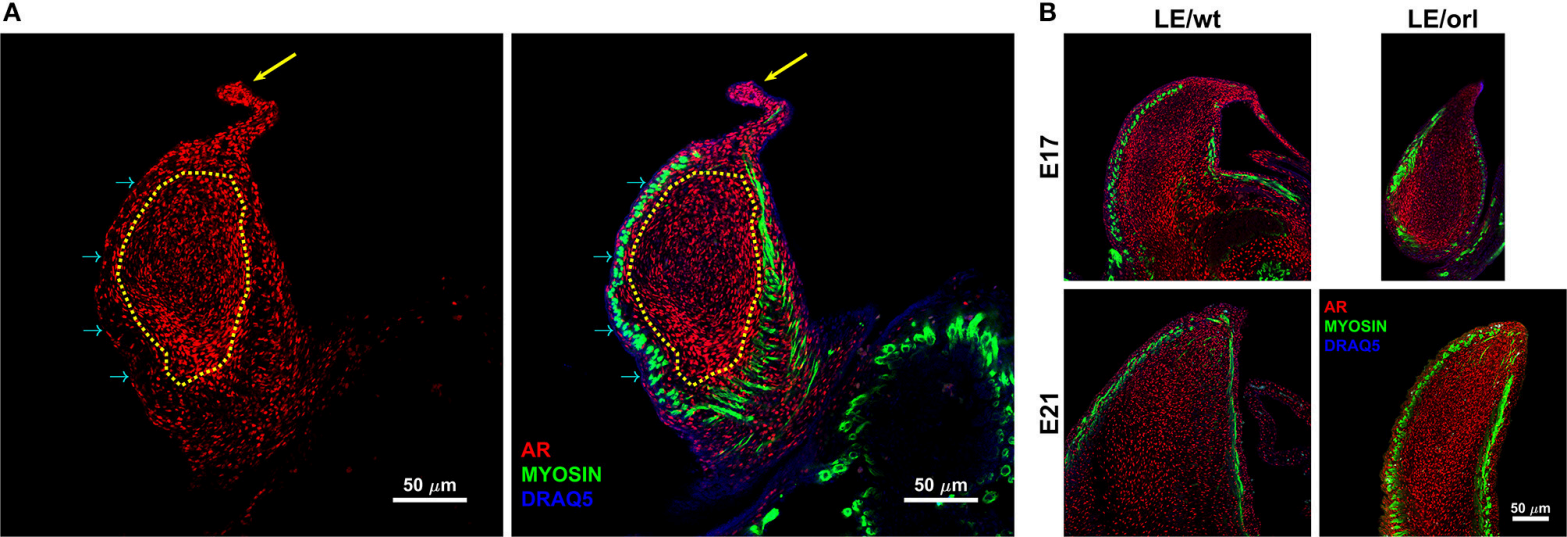
Confocal images of the sagittal midportion of LE/wt gubernacula show androgen receptor (AR) and muscle expression defined by anti-AR (red) and anti-myosin heavy chain (green) immunofluorescent staining. Images are oriented in the longitudinal plane with the cord oriented superiorly. **(A)** Representative image of E17 LE/wt gubernaculum with localization of AR visible throughout the inner mesenchymal core (traced in yellow) and the gubernacular cord (yellow arrow). As noted in the merged image (second panel), nuclear localization (magenta) is strongly evident in the tip region and cord. However, myosin positive cells are negative for AR, several prominent examples highlighted by cyan arrows. **(B)** In E17 and E21 gubernacula isolated from LE/wt and LE/orl fetal rats, AR + cells are visible within the central mesenchyme and near the tip, which contains a previously described growth center. By E21, as the gubernaculum is starting to invert, areas of clearing with reduced AR + cell density appear proximally.

We quantified AR expression in E17 and E21 LE/wt and LE/orl immunostained gubernacula (*n* = 3–9 per group) using a custom analysis algorithm formulated in MATLAB (MATLAB 2018a; Mathworks, Natick, MA). Each confocal volume was analyzed in full. Segmentation of the gubernacula was accomplished through manual elimination of any remaining the abdominal wall tissue and enhancement of the DRAQ5 channel. Briefly, DRAQ5 signal was median filtered with a 3.3 × 3.3 × 4.3 μm kernel, plane by plane rolling ball filtering (with a 66.4 μm radius) and morphological closing (with a 20 μm radius) to fill in gaps between the cells. The gubernaculum was finally segmented using hysteresis thresholding. Identification of the “tip” region was performed manually by a masked observer, and was defined as the region between the cord (arrow in Figure 1A) and the beginning of the dense muscular layer. The mesenchymal core comprised the region deep to the muscle (dotted yellow line in Figure 1A). Average AR intensity for the tip region and the rest of the gubernaculum was accomplished by taking the mean intensity value of the raw stain over the segmented volumes.

### Androgen Stimulation of Gubernaculum Cells

E19 gubernaculum pairs were dissociated overnight at 4°C in 5 mg/ml collagenase with 2.5 mM CaCl. Samples were triturated and then plated on collagen or poly-L lysine (PLL)/laminin-coated 6-well tissue culture plates. Cells were then incubated for a total of 6 days in DME/F12 containing 18% fetal calf serum (FCS), rat basic fibroblast growth factor (bFGF; 2.5 ng/ml), rat epidermal growth factor (EGF; 10 ng/ml), dexamethasone (0.4 ug/ml), and penicillin-streptomycin supplemented with 0, 0.1, 1.0, or 10.0 nM DHT. Media was changed on each of the 6 days of culture. Immunofluorescent staining of AR in gub cells was carried out using 1:200 dilution of the rabbit anti-AR antibody (Abcam cat# ab133273) in PBS at 4°C overnight. Cells were washed 3X with PBS and then incubated with donkey anti-rabbit Alexaflour 555 and Dapi (Fisher cat#EN62248) to visualize nuclei. On day 6, 5 random areas were captured from each treatment protocol using an Olympus BX-60 florescence microscope with an Evolution QEi 12-bit digital camera (Media Cybernectics) and a 20X objective. AR+ cells were counted manually using Image J software (National Institutes of Health, Bethesda, Maryland, USA, https://imagej.nih.gov/ij/).

### Membrane Organ Culture and Muscle Development Assay

Individual gubernacula were dissected and placed on Millipore membranes in a six-well dish containing 2 ml of DME/F12 media with 2% stripped FCS, ITS and pen-strep as described previously (25). Media with or without 10 nM DHT was added, and gubernacula were harvested 24 h after incubation in PBS. Conditioned media (200 μl) from individual gubernacula grown on Millipore membranes with and without DHT stimulation was removed at 24, 48, 72, and 96 h with replacement of an equivalent volume of media to the appropriate wells.

Membrane culture was also used to assay muscle development by embedding E17 gubernacula in Matrigel® for 6 days, allowing gubernaculum myoblasts to migrate peripherally and form striated muscle fibers as described previously (25). We used myosin IF to visualize individual fibers with 1:10 dilution of A4.1025, a myosin-specific monoclonal antibody (Developmental Studies Hybridoma Bank (DSHB), Iowa City, Iowa). We measured muscle fibers using the Image-Pro histogram generated masking (v.6.3, Media Cybernetics). These measurements included area, feret minimum and maximum diameter (length) and aspect, as defined by the Image-Pro program. In order to reduce background, non-fiber counts cutoffs were established for area and aspect. Every data point was tabulated in ascending order, and aspect and area measurements < 5 and 500, respectively, were deleted.

### Hyaluronan (HA) Analysis

DHT-treated gubernaculum pairs were homogenized in cell lysis buffer using a Rotor stator. The extracts were then spun at 17,800 rpm for 5 min. Supernatants were removed and stored at 4°C and the pellets were frozen at −20°C. After completion of sample collection, we measured HA levels concurrently in all available samples using R&D Biosystems Hyaluronan Quantikine ELISA Kit (cat# DHYAL0) in accordance with the manufacturer's instructions.

### Glycosaminoglycan (GAG) Analysis

Rat gubernacula were digested with 0.3 ml of 4 mg/ml Pronase (Roche, 11459643001) in PBS for 1.5 h at 37°C. Collagenase (Gibco, 17101015) 0.1 ml, 2 mg/ml in PBS, was added to digestion samples, and samples were incubated at 37°C overnight. After centrifugation at 14,000 rpm for 10 min, the enzyme solution was removed. 150 μl of 50 mM Tris-HCl (pH = 7.0) was added to the pellets, and the sample solution was sonicated on ice for 15 s, 2 times. The sample solutions were stored at −20°C before pretreatment for GAG analysis. Further, preparation of gubernaculum and conditioned media samples for GAG analysis and quantification of GAG levels by liquid chromatography tandem mass spectrometry (LC-MS/MS) was previously described (26). In brief, 40 μl of sample solution was added into a 96 well Omega 10 K filter plate (PN 8034) (Pall Co, MI) with 60 μl of 50 mM Tris-HCl (pH = 7.0) with a 96 well receiver plate at the bottom. After centrifugation for 15 min at 2,500 g, a cocktail mixture with 60 μl of 50 mM Tris-HCl (pH = 7.0), 10 μl of 200 mU/ml Heparatinase, 10 μl of 200 mU/ml keratanase II (Seikagaku Co., Japan), 10 μl of 50 mU/ml Chondroitinase B, and 10 μl of 5 μlg/ml IS (chondrosine) were added to the filter plate, and incubated overnight at 37°C to digest oligosaccharides into disaccharides. All enzymes were provided by Seikagaku Co. (Tokyo, Japan). After centrifugation for 15 min at 2,500 g, filtrated pretreatment sample was stored at −20°C before GAG analysis by LC-MS/MS. A sample aliquot of 5 μl was injected into LC-MS/MS consisting of a 1,290 Infinity LC system with a 6,460 triple quad mass spectrometer (Agilent Technologies, Palo Alto, CA). After separation of disaccharides on a Hypercarb column (2.0 mm i.d. 50 mm length; 5 μm particles; Thermo Scientific, USA), specific precursor ion and product ion were detected to quantify each disaccharide, respectively, (HS-0S 378.3; 175.1, HS-NS 416, 138; mono-sulfated KS 462, 97; di-sulfated KS 542, 462; IS 354.3, 193.1). GAG levels in rat gubernaculum was normalized by protein concentrations. The protein assay was performed using a bicinchoninic acid assay (Pierce BCA Protein Assay Kit, Thermo Fisher Scientific, Prod#23227).

### Apparent Elastic Modulus of the Fetal Gubernaculum

Isolated gubernacula were incubated in RS-I media (Aqix Ltd, London, United Kingdom) for no more than 6 h before measurement. Mechanical characterization of each intact gubernaculum was performed using micropipette aspiration on a stereoscope stage (Discovery.V8; Carl Zeiss Inc, Thornwood, NY), similar to previous descriptions (27–33). Two micromanipulators were used to position the tissue samples and the aspiration pipette. Aspiration was conducted with micropipettes freshly pulled from 1 mm thin wall capillaries (WPI, Sarasota, FL) using Flaming/Brown Micropipette Puller (P-97; Sutter Instruments, Novato, CA). Micropipettes were trimmed to diameters of approximately 80 μm using a ceramic cutting square (Sutter). For measurement, gubernacula were equilibrated in ice-cold PBS for 15 min. Suction was applied to the micropipette through a vacuum regulator at −70 kPa. Three positions were aspirated along the outer curvature of each gubernaculum: the *base* (defined as the circumferential bottom 25% of height adjacent to the pelvic floor), *midportion* (between 40 and 60% of the height), and *tip* (top 25% of the height) of the organ. Each aspiration was allowed to stabilize for 3 min to allow the tissue deformation to reach steady state and imaged with a camera mounted on the stereoscope (Canon EOS EOS T5 DSLR; Canon, Melville, NY). Apparent elastic modulus (stiffness) is defined by Equation 1 (27):

(1) $$\begin{array}{r} E_{app}=\frac{3a\Delta p}{2\pi L}\Phi \end{array}$$

Where *a* is the micropipette radius, Δ*p* is the aspiration pressure, *L* is the length of aspirated tissue, and Φ is the wall function, a parameter to account for differences in pipette geometry, given by Equation 2 (27), below, where w is the pipette wall thickness:

(2) $$\begin{array}{r} \Phi ≅\frac{1}{2}\frac{1+\frac{w}{a}}{1+\frac{w}{2a}}\ln\frac{8a}{w} \end{array}$$

### Statistical Analysis

We used ANOVA, UNIANOVA or independent sample *t* tests (Mann-Whitney) in SPSS® v25 (IBM Corporation) to test for differences between groups. Parametric or non-parametric *p* values < 0.05 were considered significant.

## Results

### Full-Length AR has a Distinct Expression Pattern in Fetal Gubernacular Mesenchyme

**Figure 2:**
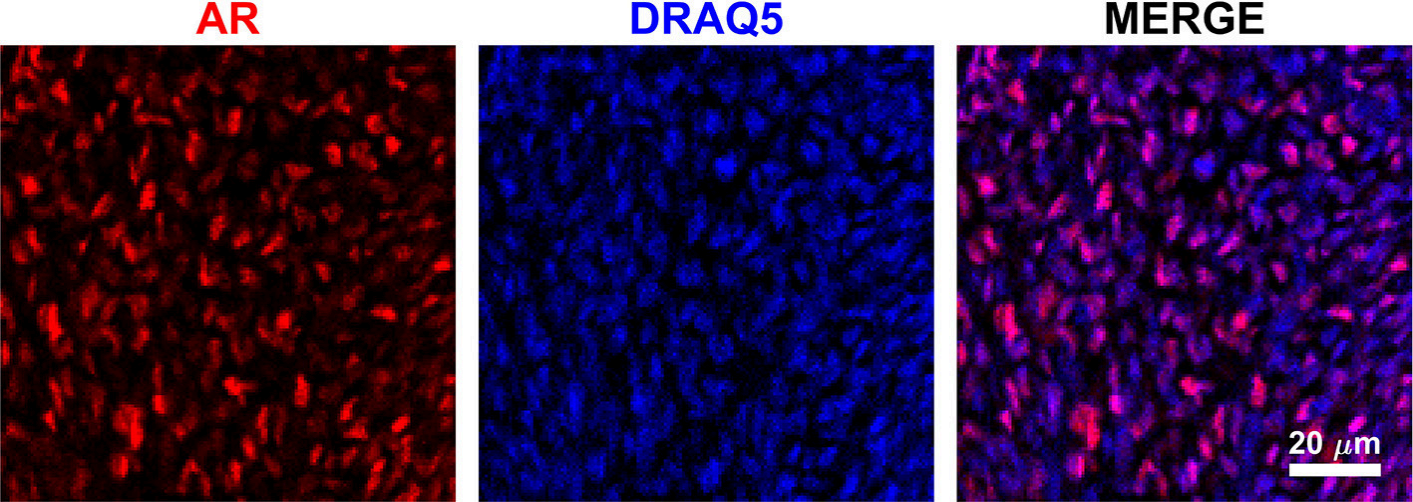
Image of gubernacular mesenchymal core in culture shows variable AR expression among cells, and predominantly nuclear localization of the receptor (red = AR, blue = DRAQ5 nuclear stain). Image shown of E17 LE/wt, localization and staining patter qualitatively conserved at E19 and E21 in both LE/wt and LE/orl.

**Figure 3:**
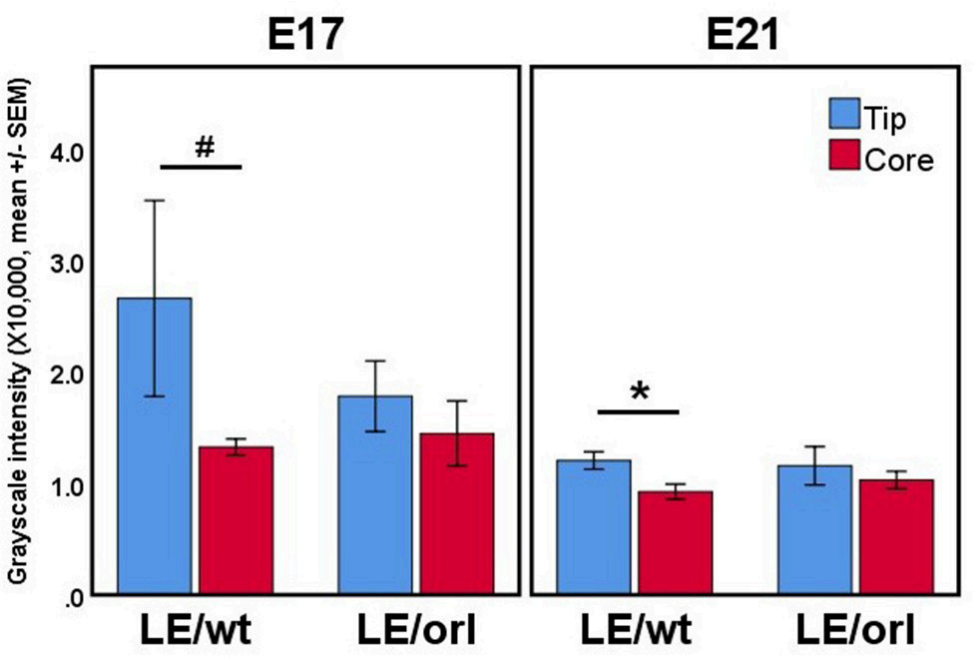
Confocal volumes of AR immunostaining in LE/wt and LE/orl gubernacula (*n* = 3–8). The volumes were segmented into “tip” regions immediately inferior to the cord, and “core” regions of the mesenchymal bulk. In all cases, the tip region trended toward increased mean staining intensity, and this trend was significant in E21 LE/wt (*p < 0.05) and approached significance in E17 LE/wt (#*p* = 0.06) analyses. We did not observe increased AR expression at the tip relative to other regions in LE/orl gubernacula.

Using a specific anti-AR antibody that recognizes the full-length form of the receptor and confocal imaging, we found that nuclear AR is expressed widely in mesenchymal cells of the fetal rat gubernaculum (Figure 1A, yellow dashed region), and is excluded from cremaster muscle cells (Figure 1A; cyan arrows). At E17 in LE/wt fetuses (Figure 1B), we observed increased AR staining intensity, which could indicate either increased cell density or increased expression levels. While we observed qualitative differences in size of the gubernacula (with LE/orl tissues being thinner), we did not observe any qualitative differences in cell density. In aggregate, the results indicate enhanced AR expression in nuclei of mesenchymal cells in the tip region and adjacent to developing cremaster muscle. AR immunofluorescence was less strongly nuclear within cells defined as a distal growth zone in studies of the postnatal rat gubernaculum (34, 35). By E21 (Figure 1B), we noted areas of decreased cellular density beneath the cremaster muscle layers. The pattern of AR expression was similar in LE/orl fetuses at E17 and E21, although a narrower organ at E21 was associated with increased density of AR+ cells (Figure 1B). A higher power view of the central mesenchyme (Figure 2) confirms variable AR expression between cells and nuclear localization of AR in this region. Quantitative analysis suggested enhanced AR expression in the tip region as compared to the mesenchymal core in LE/wt but not LE/orl gubernacula (Figure 3).

### DHT Stimulates AR Expression in Primary Gubernaculum Cells

**Figure 4:**
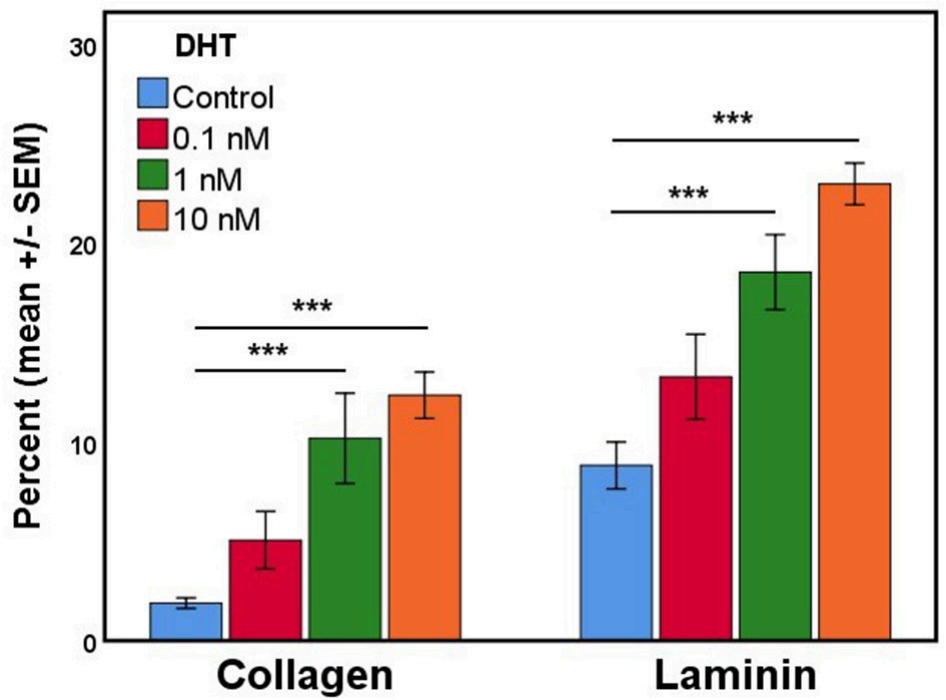
The mean percentage of cultured gubernaculum mesenchymal cells expressing AR (*n* = 6 per exposure group) is enhanced by DHT in a dose- and substrate-dependent manner (original magnification, 40X; ***p < 0.001).

In the process of developing fetal gubernaculum cell lines (25), we observed loss of AR expression over time with repeated passaging of cultured E17 gubernaculum cells, both by immunofluorescent staining and Western blotting of protein extracts. In the current experiments, we confirmed that AR+ cells in culture show varying degrees of nuclear expression of the receptor, consistent with *in vivo* distribution as shown in images of the intact mesenchymal core (Figure 2) and AR expression is enhanced in a dose-dependent fashion by DHT stimulation (Figure 4). This effect was highly significant (*p* < 0.001 by ANOVA) for cells grown on either collagen type I or PLL/laminin substrata, but the response was more robust for PLL/laminin.

### DHT does not Affect the Morphology of Gubernacular Muscle Cells in a Developmental Assay

**Figure 5:**
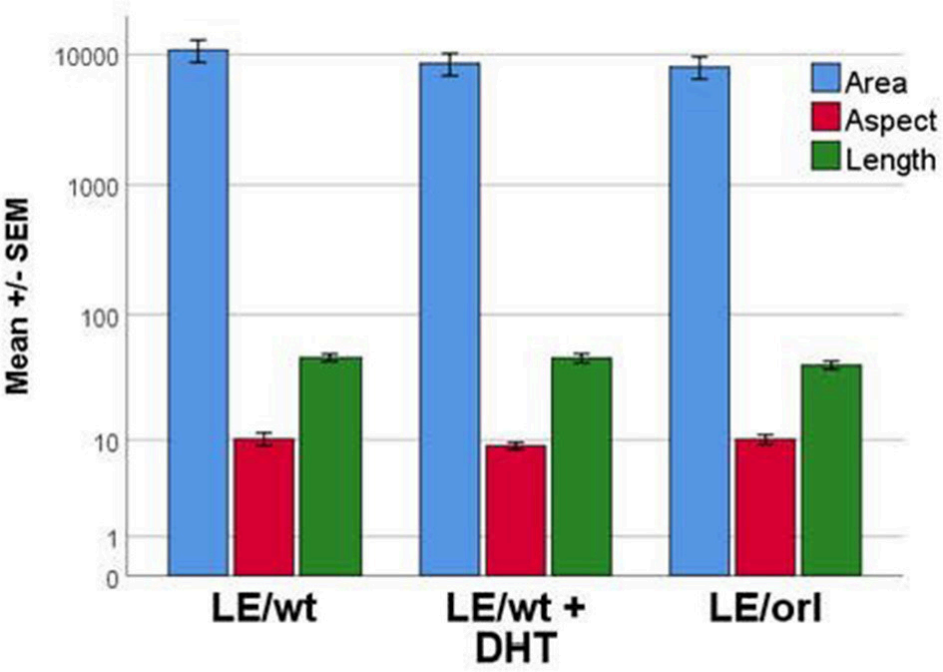
Muscle cell morphology was assessed *in vitro* with fetal gubernacula immobilized in Matrigel to allow peripheral migration of myoblasts and subsequent formation of striated muscle fibers. Using this model, we found no differences in muscle cell morphology between LE/wt samples exposed (*n* = 10) and unexposed (*n* = 10) to 10 nM DHT and unexposed LE/orl (*n* = 19) fetal gubernaculum cells following cell migration and muscle fiber formation (results expressed using a logarithmic scale; area in μm^2^; aspect and length in μm).

We previously reported a fetal gubernaculum muscle development organ culture model, which utilizes intact E17 gubernacula embedded in Matrigel on PLL-coated plates (25). In this model, muscle precursors migrate away from the organ to form striated muscle fibers. Using this assay, we found no significant effect of DHT on muscle morphometry following 6 days of LE/wt gubernaculum culture, nor any significant differences in muscle fiber size between LE/wt and LE/orl samples (Figure 5). Although the assay could not provide accurate quantitation of developing muscle fibers, we found no other qualitative strain- or hormone-dependent differences in muscle development.

### DHT does not Stimulate Production of Extracellular Matrix Proteins

**Figure 6:**
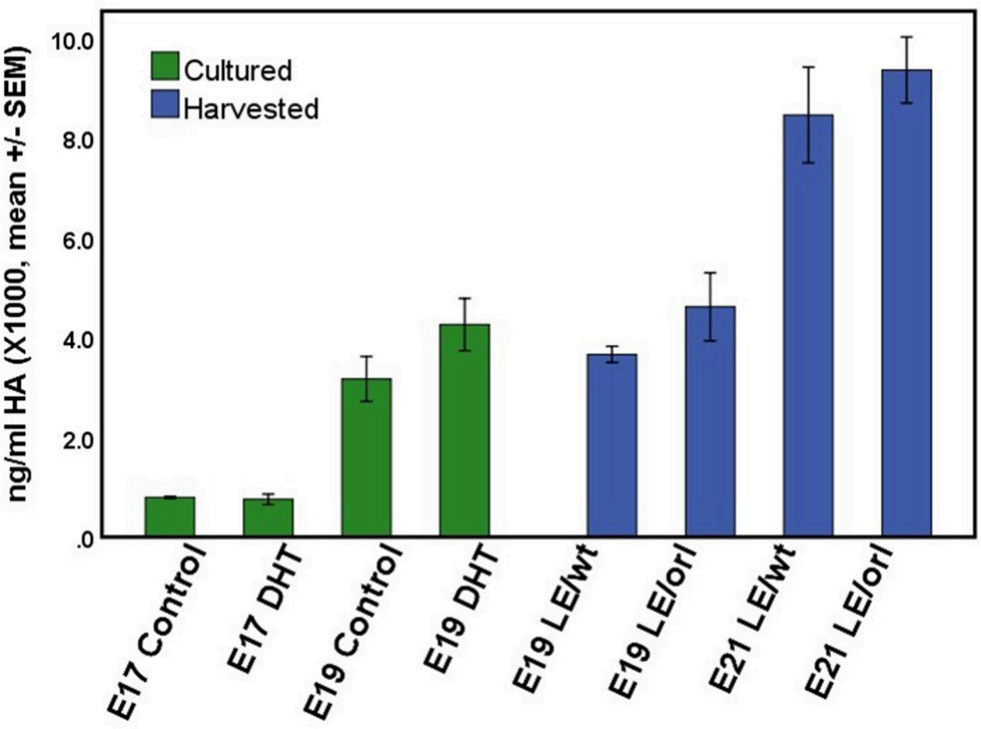
HA concentration did not differ significantly between LE/wt E17 or E19 gubernacula cultured with and without exposure to 10 nM DHT (green bars; *n* = 5–6 per treatment group per time point) or between LE/wt and LE/orl gubernacula harvested at E19 or E21 (blue bars; *n* = 4 per strain per time point).

Using a sensitive rat-specific ELISA assay, we found that rat HA is easily measurable in the fetal gubernaculum, but levels are not influenced by exposure to DHT (Figure 6). Similarly, we found no significant differences in HA levels between LE/wt and LE/orl gubernacula at E19 or E21, during the outgrowth phase of the organ.

**Figure 7:**
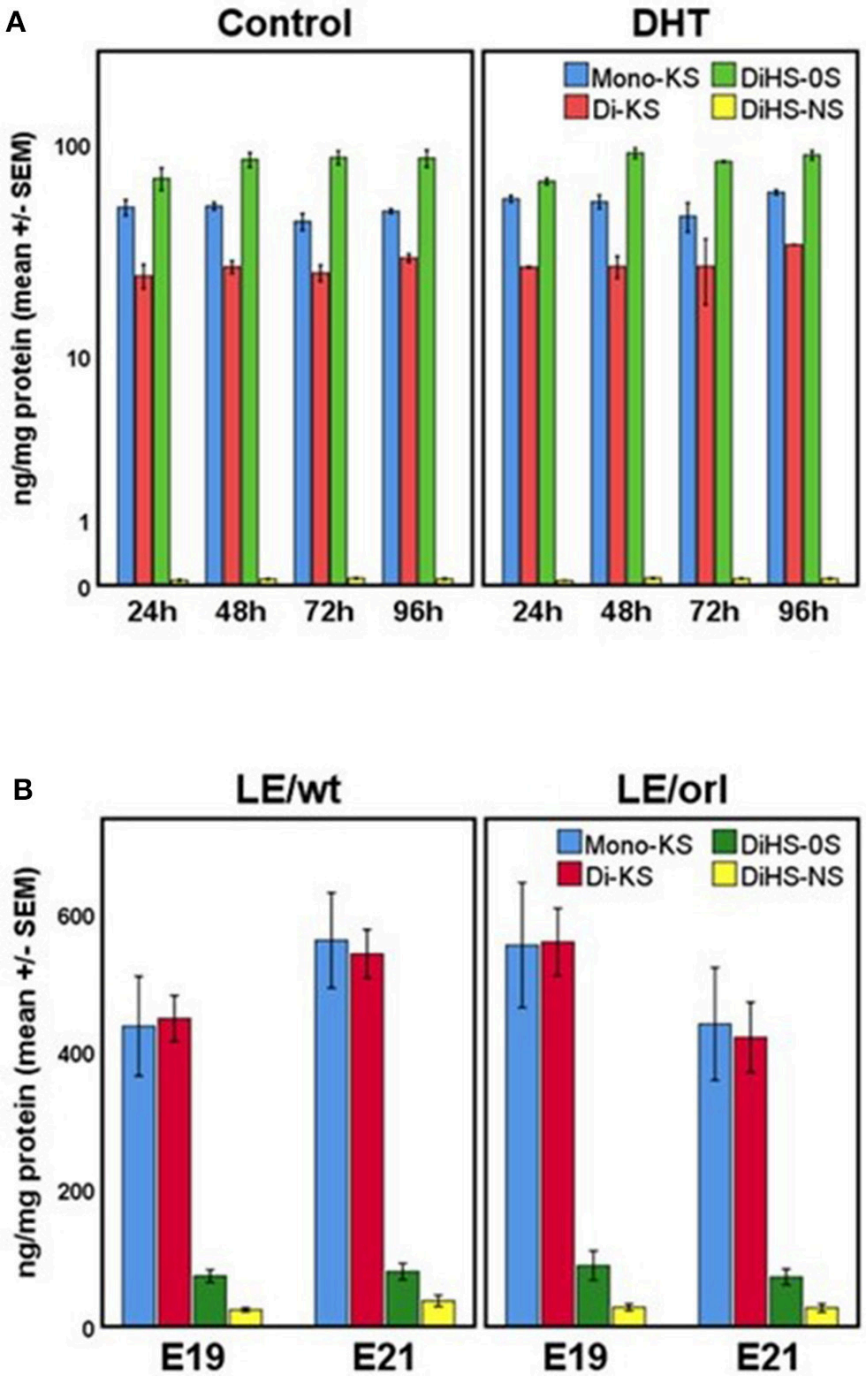
**(A)** GAG concentrations in conditioned media from LE/wt gubernaculum membrane cultures show no differences between control and DHT-exposed samples (results expressed using a logarithmic scale; *n* = 2–3 per treatment group per time point). **(B)** Gubernaculum GAG expression revealed increasing mono- and di-keratan sulfate (Mono-KS, Di-KS) concentrations between E19 and E21 in LE/wt with a reverse trend in LE/orl fetuses, although differences between strains were not significant (*n* = 10–18 per strain per time point).

Our sensitive tandem mass spectrometry (LC-MS/MS) assay results (Figure 7) suggest that heparan sulfate (DiHS-NS, DiHS-0S) and mono- and di-keratan sulfate (DS-KS, MS-KS) levels are measurable and reproducible in fetal gubernaculum tissue and conditioned media. In membrane whole mount cultures, we measured GAG secretion into the media that exceeded baseline values, but did not observe any effect of DHT (Figure 7A). The concentration of the most abundant GAG, KS, is maintained in LE/wt between E19 and E21, but reduced (although not significantly) by E21 in LE/orl gubernaculum (Figure 7B).

### Stiffness is Maximal in the Gubernacular Midportion in LE/wt but not LE/orl Organs

**Figure 8:**
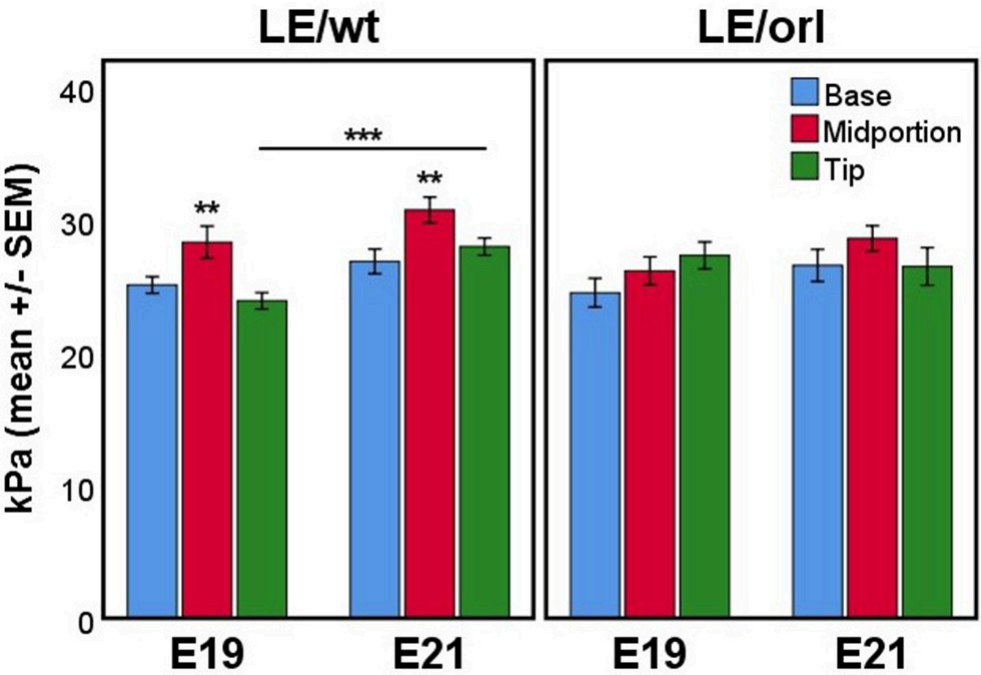
Micropipette aspiration (*n* = 13–17 per strain at each time point) showed increased stiffness of the midportion relative to both base and tip at E19 and E21 in LE/wt gubernaculum (**p < 0.01, ANOVA) and a significant increase in stiffness at the tip between E19 and E21 (***p < 0.001, independent sample *t* test). Significant differences in stiffness between gubernaculum segments or gestational days was not observed in the LE/orl gubernaculum.

Using micropipette aspiration, we determined the apparent elastic modulus (*E*_*app*_, in kilopasquales, kPa) of the fetal gubernaculum at E19 and E21. To account for potential spatial variation, we measured 3 locations along the outer curvature: base (near the pelvic floor), midportion, and tip (near the gubernacular cord); additionally we completed measurements at E19 and E21 to capture changes in mechanics as the gubernaculum undergoes developmental remodeling. In LE/wt rat fetuses, we observed increased stiffness in the midportion at both E19 and E21 compared to the other regions measured (*p* < 0.01), and increasing stiffness between E19 and E21 (*p* < 0.001; Figure 8). In contrast, LE/orl fetal gubernacula did not show regional differences in stiffness or significant changes with increasing gestational age.

## Discussion

The results of these studies are consistent with prior work defining mesenchymal rather than muscle cells as targets of androgens in the fetal gubernaculum (14, 16), cell which appear to contribute to formation of the cremaster muscle (25). Loss of AR expression once mesenchymal cells have differentiated into cremaster muscle may explain why conditional *Ar* deletion in cells that have already undergone myogenic commitment does not interfere with testicular descent (5). Enhanced nuclear AR expression in the tip and cord of the gubernaculum is also consistent with prior studies showing that failure of the cord to shorten and delayed or absent inversion of the gubernaculum occur with loss of AR signaling (36, 37). The present data further indicate that AR is expressed in the gubernaculum during a time frame that overlaps with susceptibility of the rat fetus to antiandrogen-induced cryptorchidism. Our prior expression profiling data showed that *Ar* transcript levels are suppressed in gubernacula exposed to DHT (10 or 30 nM for 24 h) *in vitro* (38), while the current studies show dose-dependent increased expression of AR protein in dispersed gubernacular mesenchymal cells after exposure to the same DHT concentration (10 nM) for 7 days. This may be due to differing durations of exposure in each experiment, although divergent responses of mRNA and protein to the same stimulus may occur, particularly in cases of transcript suppression or when analyzing whole tissues that contain different cell types (39). Nevertheless, the observation that androgen is able to regulate expression of its receptor suggests that gubernacular AR is functional, and that the effect of androgens in stimulating testicular descent has an innervation-independent mechanism.

The role of androgens in each phase of testicular descent remains unclear. Available data do not consistently support the theory that regulation of transabdominal (Phase 1) and transinguinal (Phase 2) descent by INSL3 and androgens, respectively, is distinct (11). The majority of rats exposed to the antiandrogen flutamide during Phase 1 have undescended, and sometimes perirenal testes, while flutamide exposure during Phase 2 does not inhibit testicular descent (40–43). We found consistent AR expression in the gubernacular cord, also known as the cranial suspensory ligament (CSL). This finding is consistent with evidence from prior expression studies and from rodent models of AR deficiency in males and androgen exposure in females, which suggest that shortening of the cord is androgen dependent (7, 10, 37, 44–47). INSL3 and DHT regulate many of the same E17 gubernacular transcripts *in vitro* and both target WNT signaling, but the effects of DHT occur after 24 h, but not 6 h, of stimulation (38). A “delayed secondary response” (36, 48) to AR signaling may occur via crosstalk with other signaling pathways, and could explain why the gubernaculum is less sensitive to antiandrogens than other fetal reproductive organs (49, 50).

The current experiments suggest that androgens do not have a major role in stimulating production of CM or defining muscle fiber size in the rat gubernaculum. These results are consistent with observations that swelling of the gubernaculum still occurs to some extent in mice with spontaneous (*Tfm*) or transgenic (ARKO) *Ar* inactivation, or in the LE/orl rat, which shows reduced fetal androgen synthesis and action (10, 21, 37). Swelling of the gubernaculum affects its shape, and likely its mechanical properties and function. ECM components facilitate swelling due to osmotic pressure but are also involved in extracellular signaling pathways and cellular differentiation (51). In larger mammals such as pig and man, the gubernaculum consists primarily of mesenchymal cells embedded in ECM. In these species, ECM deposition and degradation are thought to drive Phase 1 and 2 of testicular descent, respectively (52–54). The GAG content of the fully developed porcine fetal gubernaculum consists of dermatan sulfate (50%), hyaluronic acid (30%), and chondroitin/keratan and heparan sulfates (10% each) (54). While there are no prior studies for comparison, our data suggest that KS is the most abundant GAG in rat fetuses, possibly because muscle is a much more prominent component of the rodent gubernaculum (34).

A known role for androgen and INSL3 is stimulation of cellular proliferation via additive effects that were demonstrated using the *in vitro* model employed in the current study (17, 18). We previously reported data suggesting that proliferating myogenic precursors contribute to enlargement of the mesenchymal core and cremaster muscle, and that gubernacular mesenchymal cells exhibit characteristics of myofibroblasts (25), a cell type that produces ECM and contributes to tissue remodeling (55). If the main function of androgens is to stimulate cellular proliferation in the gubernaculum, we would not anticipate relative changes in GAG or HA concentrations following exposure to DHT.

Our genetic and genomic studies of the LE/orl rat, a model of inherited cryptorchidism, suggest that dysfunctional AR signaling contributes to failure of testicular descent in this strain. In both LE/orl and flutamide-exposed rats, undescended testes become misdirected and reside in the superficial inguinal pouch (21, 36, 41). Testosterone levels and muscle-specific transcript levels are reduced, DHT-responsive transcripts and cremaster muscle patterning are altered, and gubernacular inversion is delayed or abnormal in LE/orl fetuses (21, 23, 56). We used linkage analysis and whole genome sequencing of the LE/orl strain (22) to identify homozygous variants in *Syne2* and *Ncoa4*, two genes encoding AR-interacting proteins that are also implicated in myogenesis (57–60). Our data suggest genetic interaction between *Syne2* and *Ncoa4*, and by selective breeding to enrich for LE/orl *Syne2* and *Ncoa4* alleles in outbred strains, we were able to reproduce the cryptorchid phenotype (22). The muscle defects and altered shape of the fetal LE/orl gubernaculum (21) are consistent with the abnormal elastic modulus that we identified in the present studies. Consistent with a role for abnormal AR signaling in the LE/orl strain, the present data suggest that neither DHT nor the LE/orl background appeared to influence muscle fiber morphology or ECM composition in fetal gubernacula.

Our data suggest regional differences in stiffness of the gubernaculum characterized by peak stiffness of the midportion, and increasing stiffness at the tip between E19 and E21. The reduced swelling that we observed in the LE/orl gubernaculum herein and in prior studies (21, 23) may account for differences in its biomechanical properties. In a prior study using histological sections, matrix metalloproteinase (MMP)-expression was reported in cells adjacent to the inverting perinatal rat gubernaculum, suggesting that active ECM remodeling may facilitate gubernacular motility (61). Areas of clearing that we observed in the region between the central mesenchyme and peripheral muscle layers may be comparable to previous anatomical studies of the human fetus, which revealed loosening of the connections between the gubernaculum and surrounding cremaster-lined inguinal canal just prior to testicular descent (62). The increasing elastic modulus of the gubernaculum at the midportion and tip, along with symmetrical detachment of the mesenchymal core from the surrounding muscle, may enable force generation in a scrotal direction and transinguinal passage of the testis. Swelling of the mesenchymal core is subtle in rodents but if insufficient, may affect the shape and mechanical properties of the gubernaculum and its capacity for correct inversion.

In summary, current and prior data suggest that androgens enhance AR expression and proliferation of mesenchymal cells of the fetal gubernaculum, and that AR expression is lost with myogenic differentiation. Androgens may also have indirect effects on ECM production by stimulating the growth of AR-expressing myofibroblasts. Susceptibility to cryptorchidism in the LE/orl rat is associated with altered stiffness, but not with changes in ECM composition or muscle fiber morphology of the fetal gubernaculum. Regional differences in stiffness and localized ECM remodeling may be required for successful transinguinal passage of the gubernaculum and testis.

## Disclaimer

This work was prepared while Dr. Julia Barthold was employed at Nemours. The opinions expressed in this article are the author's own and do not reflect the view of the National Institutes of Health, the Department of Health and Human Services, or the United States government.

## Ethics Statement

This study was carried out in accordance in a facility accredited by the Association for Assessment and Accreditation of Laboratory Animal Care International, based on the Guide for the Care and Use of Laboratory Animals. The protocol was approved by the Nemours Animal Care and Use Committee.

## Author Contributions

All authors listed have made a substantial, direct and intellectual contribution to the work, and approved it for publication. JM and JG performed mechanical testing and quantitative image analysis. AR, AM, and DG performed dissection, cell and organ culture, and staining experiments. KS and ST performed matrix analysis. JSB, AR and JM were responsible for drafting the manuscript. All authors contributed to the development and interpretation of the experimental data as well as manuscript editing.

### Conflict of Interest Statement

The authors declare that the research was conducted in the absence of any commercial or financial relationships that could be construed as a potential conflict of interest.
